# Multiple transcription factors co-regulate the *Mycobacterium tuberculosis* adaptation response to vitamin C

**DOI:** 10.1186/s12864-019-6190-3

**Published:** 2019-11-21

**Authors:** Malobi Nandi, Kriti Sikri, Neha Chaudhary, Shekhar Chintamani Mande, Ravi Datta Sharma, Jaya Sivaswami Tyagi

**Affiliations:** 1 Department of Biotechnology, All India Institute of Medical Sciences, Ansari Nagar, New Delhi, 110029 India; 2 Amity Institute of Biotechnology, Amity University, Manesar, Haryana 122413 India; 3 Present address: Department of Biochemistry and Molecular Biology, University of Nebraska Medical Center, Omaha, NE USA; 4 National Centre for Cell Science, Pune, Maharashtra 411007 India; 5 Translational Health Science and Technology Institute, Faridabad, Haryana 121001 India

**Keywords:** Mtb-TRN (Mtb-transcriptional regulatory network), Dormancy, Vitamin C, Transcriptome, *Mycobacterium tuberculosis*

## Abstract

**Background:**

Latent tuberculosis infection is attributed in part to the existence of *Mycobacterium tuberculosis* in a persistent non-replicating dormant state that is associated with tolerance to host defence mechanisms and antibiotics. We have recently reported that vitamin C treatment of *M*. *tuberculosis* triggers the rapid development of bacterial dormancy. Temporal genome-wide transcriptome analysis has revealed that vitamin C-induced dormancy is associated with a large-scale modulation of gene expression in *M. tuberculosis*.

**Results:**

An updated transcriptional regulatory network of *M.tuberculosis* (Mtb-TRN) consisting of 178 regulators and 3432 target genes was constructed. The temporal transcriptome data generated in response to vitamin C was overlaid on the Mtb-TRN (vitamin C Mtb-TRN) to derive insights into the transcriptional regulatory features in vitamin C-adapted bacteria. Statistical analysis using Fisher’s exact test predicted that 56 regulators play a central role in modulating genes which are involved in growth, respiration, metabolism and repair functions. Rv0348, DevR, MprA and RegX3 participate in a core temporal regulatory response during 0.25 h to 8 h of vitamin C treatment. Temporal network analysis further revealed Rv0348 to be the most prominent hub regulator with maximum interactions in the vitamin C Mtb-TRN. Experimental analysis revealed that Rv0348 and DevR proteins interact with each other, and this interaction results in an enhanced binding of DevR to its target promoter. These findings, together with the enhanced expression of *devR* and *Rv0348* transcriptional regulators, indicate a second-level regulation of target genes through transcription factor- transcription factor interactions.

**Conclusions:**

Temporal regulatory analysis of the vitamin C Mtb-TRN revealed that there is involvement of multiple regulators during bacterial adaptation to dormancy. Our findings suggest that Rv0348 is a prominent hub regulator in the vitamin C model and large-scale modulation of gene expression is achieved through interactions of Rv0348 with other transcriptional regulators.

## Background

The existence of *Mycobacterium tuberculosis* (Mtb) in a state of dormancy/persistence during latent tuberculosis (TB) infection in immunocompetent individuals poses a significant barrier in eradication of this pathogen. Dormant/persistent TB bacteria adapt to a physiologically altered state in response to host-derived stresses and are not easily cleared by the existing TB drugs. Various in-vitro models of dormancy/persistence have provided valuable insights into the signature responses adopted by Mtb for survival under stress conditions [1–8]. Vitamin C (vit C) has been shown to trigger Mtb dormancy by O_2_ depletion, leading to a rapid induction of DevR and its regulon genes [9]. The bacterial response to vit C exhibits a significant overlap in gene expression with the other dormancy models and hence, is suggested to be a multi-stress model to study Mtb dormancy adaptation mechanisms [10] .

Genome-wide transcriptome studies have provided significant insights into gene regulatory mechanisms and pathways utilised by bacteria during adaptation to dormancy [11–20]. Two-component systems and sigma factors such as DevR, MprA, PhoP, RegX3, SigB, SigH and SigE, are the best characterized regulators of Mtb that mediate survival under various stresses [13, 21–27]. The induction of DevR and its regulon genes serves as the classic signature of Mtb under hypoxia and nitrosative stress [3, 13]. PhoP, MtrA, MprA and RegX3 are known to be actively involved in functions like virulence, regulation of WhiB proteins and complex lipid biosynthesis, cell envelop stress response, persistence and sigma factor regulation, phosphate uptake, and aerobic respiration [12, 28–31].

Previously, our laboratory reported that vit C-induced dormancy in Mtb is associated with bacterial growth stasis, progression to viable but non-culturable (VBNC) state, loss of acid-fastness and reduction in length, protective response to oxidative stress, lipid metabolism and modulation of anti-tuberculosis drugs [32]. To decipher the key regulators of vit C-induced dormancy, we have built a comprehensive Mtb transcriptional regulatory network (Mtb-TRN) that includes all possible regulator-target interactions of Mtb H37Rv. This network was overlaid with microarray gene expression data generated from vit C-treated Mtb (GEO accession no: GSE101048 [32]). Here we show that 56 regulators are involved in governing these extensive changes in Mtb in response to vit C treatment. We also predict Rv0348 to be the hub regulator acting via protein-protein interactions with other regulators, exemplified by interaction between Rv0348 and DevR.

## Results

### The updated transcriptional regulatory network of *Mycobacterium tuberculosis*

**Fig. 1:**
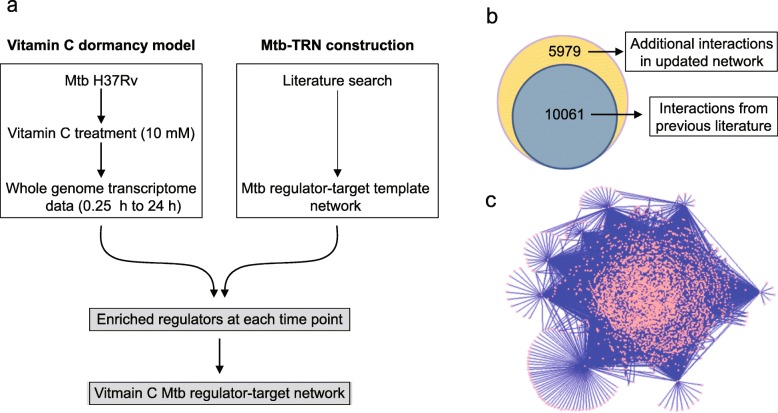
**a** A systematic flow of the protocol used for the regulator-target analysis of Mtb in response to vit C. **b** Venn diagram showing the total number of regulator-target interactions in the Mtb-TRN, where the inner grey circle shows 10,061 interactions from previous available literature and the outer yellow circle shows additional 5979 interactions from the new updated Mtb-TRN. **c** Network view of updated Mtb-TRN, wherein nodes are coloured in pink and edges (links) are coloured in blue

Firstly, we compiled the available data from multiple sources (as described in methods) to generate a Mtb transcriptional regulatory network (Mtb-TRN, Fig. 1a). This regulatory network consisted of 178 transcription factors (annotated and probable regulatory genes, two-component systems and sigma factors) with 3114 nodes (genes) and 10,061 interactions (links). Thereafter, we expanded the network by mining of literature between June 2012 to August 2018 and also performed an operon-based expansion as described [33] to include all polycistronic genes as targets of regulators. This resulted in identifying 334 new nodes and 5979 new interactions (Fig. 1b). Therefore, the final updated Mtb-TRN consists of 178 regulators with 3448 nodes and 16,040 interactions (Additional file 1: Table S1). The updated Mtb-TRN is shown in Fig. 1c.

### Multiple regulators govern the temporal adaptation response to vit C

In the present manuscript, we describe the regulatory mechanisms underlying the Mtb adaptation response to vit C. For this, previously generated microarray expression data from vit C-treated Mtb cultures [32] was re-analysed for temporal changes in gene expression. Previously, the data was analysed for co-expression using Weighted Gene Co-expression Network Analysis and 67% (*N* = 2711) of the Mtb genome was identified; whereas in the present analysis, we have identified 2543 genes to be differentially regulated (DRGs) at at-least one time point (Additional file 2: Figure S1). Genes were considered DRGs if they were up- or down-regulated by at least 2-fold in a 95% data confidence interval, *p*value and FDR value of ≤0.05. This analysis revealed an extensive remodelling of gene expression which accounted for ~ 64% of the genome. Hierarchical clustering analysis of the DRGs revealed 3 expression patterns namely, core induced (Cluster I), core repressed (Cluster II) and late time-point induction (Cluster III, Additional file 2: Figure S1).

**Fig. 2:**
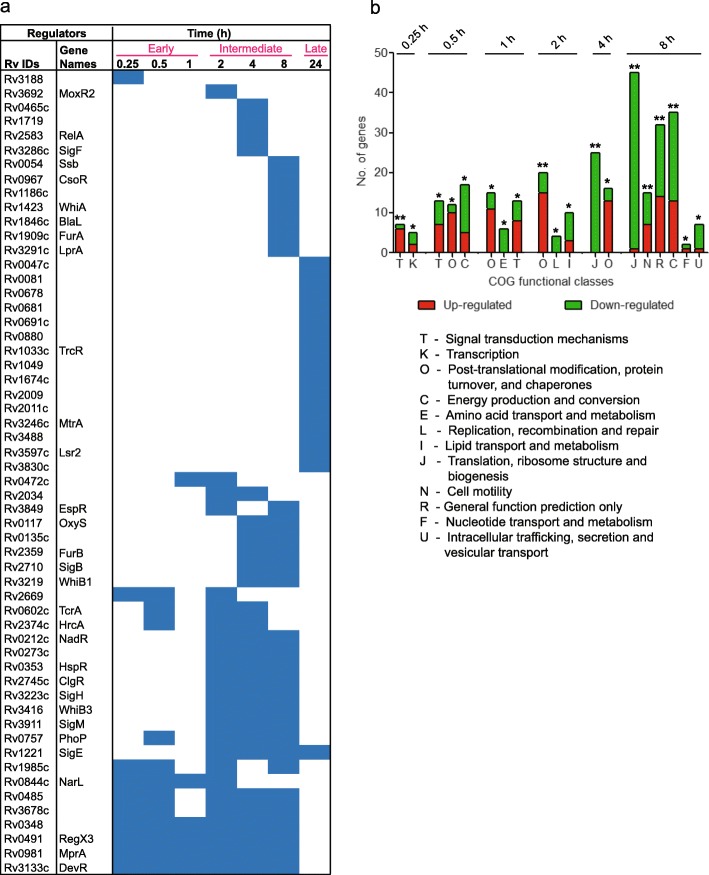
**a** Temporal enrichment of regulators. Regulators enriched at each time point (FDR corrected *p*value ≤ 0.05) are coloured in blue. The Early (0.25 to 1 h), Intermediate (2 to 8 h) and Late (24 h) time points are marked in pink. **b** COG functional enrichment of targets of the enriched regulator. COG functional categories enriched at each time point are shown. **, FDR corrected *p*value ≤ 0.05; *, *p*value ≤ 0.05 (without FDR correction)

The updated Mtb-TRN together with the DRGs were used to decipher the regulatory mechanisms underlying Mtb adaptation response to vit C, wherein all 178 regulators were subjected to enrichment analysis by overlaying the DRGs expression data on to the targets of the Mtb-TRN (described in Methods). Three temporal regulatory patterns were observed: an Early response which comprised of 0.25 h, 0.50 h and 1 h; an Intermediate response which comprised of 2 h, 4 h and 8 h; and a Late response at 24 h (Fig. 2a). Notably, 4 regulators were enriched at 6 time points starting from 0.25 h to 8 h, namely Rv0348, MprA, DevR and RegX3, which suggests their consistent role in vit C adaptation mechanisms. In addition to these regulators, the Early response was mediated by TcrA, HrcA, NarL and Rv0485 regulators (Fig. 2a). The involvement of regulators increased during the Intermediate response and consisted of OxyS, FurB, WhiB1, TcrA, HrcA, HspR, WhiB3, PhoP, NarL, Rv0485 and others (Fig. 2a). Notably, a clear switch in the regulatory response was observed at 24 h; the Late time point response comprised of 15 uniquely enriched regulators that included Lsr2, Rv0081, Rv0678, TrcR and Rv0047 (Fig. 2a). Sigma factors (SigB, SigH, SigF, SigM and SigE) were significantly enriched during the Intermediate or Late responses, suggesting a role for selectively transcribed genes in mediating the bacterial response to vit C.

### Four major functions are modulated by the enriched regulators in response to vit C

COG functional class enrichment analysis was performed on the DRG target genes of the enriched regulators using FET, wherein functional classes with *p*value ≤ 0.05 were considered enriched. The enriched COG classes that were prominently down-regulated (> 60% genes) are functions related to growth, respiration, metabolism and secretion. Only 1 enriched COG functional class was notably up-regulated (> 97% genes), namely “Post-translational modification, protein turnover, and chaperones” (Fig. 2b).

The growth-related COG functions, namely “Translation, ribosomal structure and biogenesis”, “Transcription” and “Replication, recombination and repair”, were down-regulated in the Early and Intermediate time points, indicating a comparatively low requirement of mRNA and protein synthesis during dormancy adaptation. The down-regulated genes include those encoding 30S and 50S ribosomal proteins (*rpsJ-rpsQ, rplN-rpsN, rpsH-rpmD*), which were prominently regulated by RegX3, Rv0348, FurB, WhiB1, WhiA, SigB, SigE, and SigF (Additional file 1: Table S2). Among these, RegX3, Rv0348 and FurB are known to regulate these functions under various stresses such as hypoxia and phosphate [12, 14, 19, 38, 39].

The COG function “Energy production and conversion” was also enriched at the Early and Intermediate time points and included genes involved in aerobic respiration (*nuoH-N, nuoC-G, and atpC-H*) that were down-regulated. PhoP, MprA and DevR two-component systems and Rv0348 and CsoR were identified as putative regulators regulating these functions (Additional file 1: Table S2). Among these, PhoP, MprA and DevR are associated with the down-regulation of *nuo* and other genes of respiration under various stresses [13, 20, 28, 40]. CsoR is predicted to be a key regulator in hypoxia [41] and also shown to be involved in copper homeostasis, intra-cellular survival and virulence of Mtb [42].

The genes involved in “Lipids transport and metabolism”, “Amino-acid transport and metabolism” and “Nucleotide transport and metabolism” were significantly enriched in the Intermediate time points and were down-regulated. Prominent regulators involved in this response include RegX3, PhoP, TcrA, Rv0273c, Rv1985c, Rv0234, MprA, SigE and SigM.

The only notable up-regulated functional class was “Post-translational modification, protein turnover, and chaperones” and included genes encoding several heat shock proteins (Additional file 1: Table S2), that play an important role in the pathogenesis of Mtb [43–45]. The prominent regulators that participate in this response included WhiB3, DevR, HrcA, HspR, RegX3, Rv2034, OxyS, SigB, SigE and SigH. WhiB3 is reported to respond to acidic pH, oxidative and nitrosative stresses, suggesting a significant role in regulating redox balance, persistence and granuloma formation [46–48]. The genes *groES* and *groEL2* are targets of HrcA repressor [11], which is believed to play an important part in Mtb during hypoxia [14]. Several genes encoding proteins with chaperone functions, namely, *dnaK*, *grpE*, *hsp*, *groEL1*, *groEL2*, *groES*, *clpB*, *trxC* and others (Additional file 1: Table S2) were induced in response to WhiB3, OxyS, and sigma factors SigB, SigE and SigH that are reported to be key mediators of the oxidative stress response in Mtb [32, 49].

A large number of genes (1777genes) were differentially regulated at 24 h post-vit C treatment (Additional file 2: Figure S1); however, no functional class satisfied the FET statistical threshold. Therefore, DRG targets of the enriched regulators were assigned to various COG functional classes (Additional file 2: Figure S2). Maximum targets belonged to the functions: “Transcription”, “Energy production and conversion”, “Lipid transport and metabolism”, “Amino acid transport and metabolism” and “Translation, ribosomal structure and biogenesis”. These functions were prominently regulated by Rv0081, Lsr2, TrcR, SigE and Rv0678. Rv0081 was previously shown to serve as a hub regulator during hypoxia [41, 50, 51].

### Rv0348, DevR, MprA, Lsr2, Rv0081 and Rv0678 are regulatory hubs in Mtb vit C-induced dormancy response

**Fig. 3:**
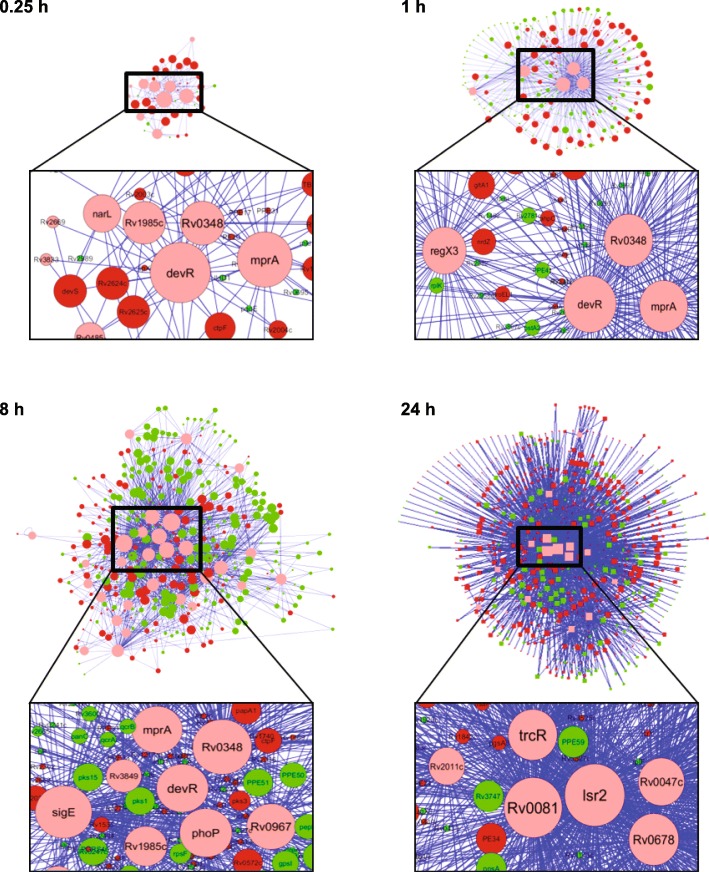
Temporal network analysis of enriched regulators and their DRG targets in vit C Mtb-TRN. Representative time points from the temporal response are shown. The regulator nodes are indicated in pink, the up-regulated target gene nodes are indicated in red, while the down-regulated target gene nodes are indicated in green. The size of the node depends upon its connectivity (links) in the network

All enriched regulators and their DRG targets were subjected to a temporal network analysis using Cytoscape’s NetworkAnalyzer function. The sizes of the nodes in the network are proportionate to their degree scores (Fig. 3 and Additional file 1: Table S3). Rv0348, MprA, DevR and RegX3 were consistently enriched from 0.25 h to 8 h (Fig. 2a). Of these, Rv0348, MprA and DevR were assigned as hub regulators based on their high degree scores (among top 5 scoring nodes) with also high betweenness centrality scores (among top 10 scoring nodes) (Fig. 3 and Additional file 1: Table S3), suggesting their central role in regulating Mtb adaptation to vit C up to 8 h. At 24 h, Lsr2, Rv0081 and Rv0678 were found to be prominent hub regulators by network analysis (Fig. 3 and Additional file 1: Table S3).

The microarray data for hub regulators involved in adaptive response up to 8 h (*Rv0348*, *mprA*, *devR*, *regX3*) and at 24 h (*lsr2, Rv0081 and Rv0678*) was confirmed by RT-qPCR. A comparable trend in expression of *Rv0348*, *devR, regX3, lsr2* and *Rv0081* was observed by both techniques (Additional file 2: Figure S3).

**Fig. 4:**
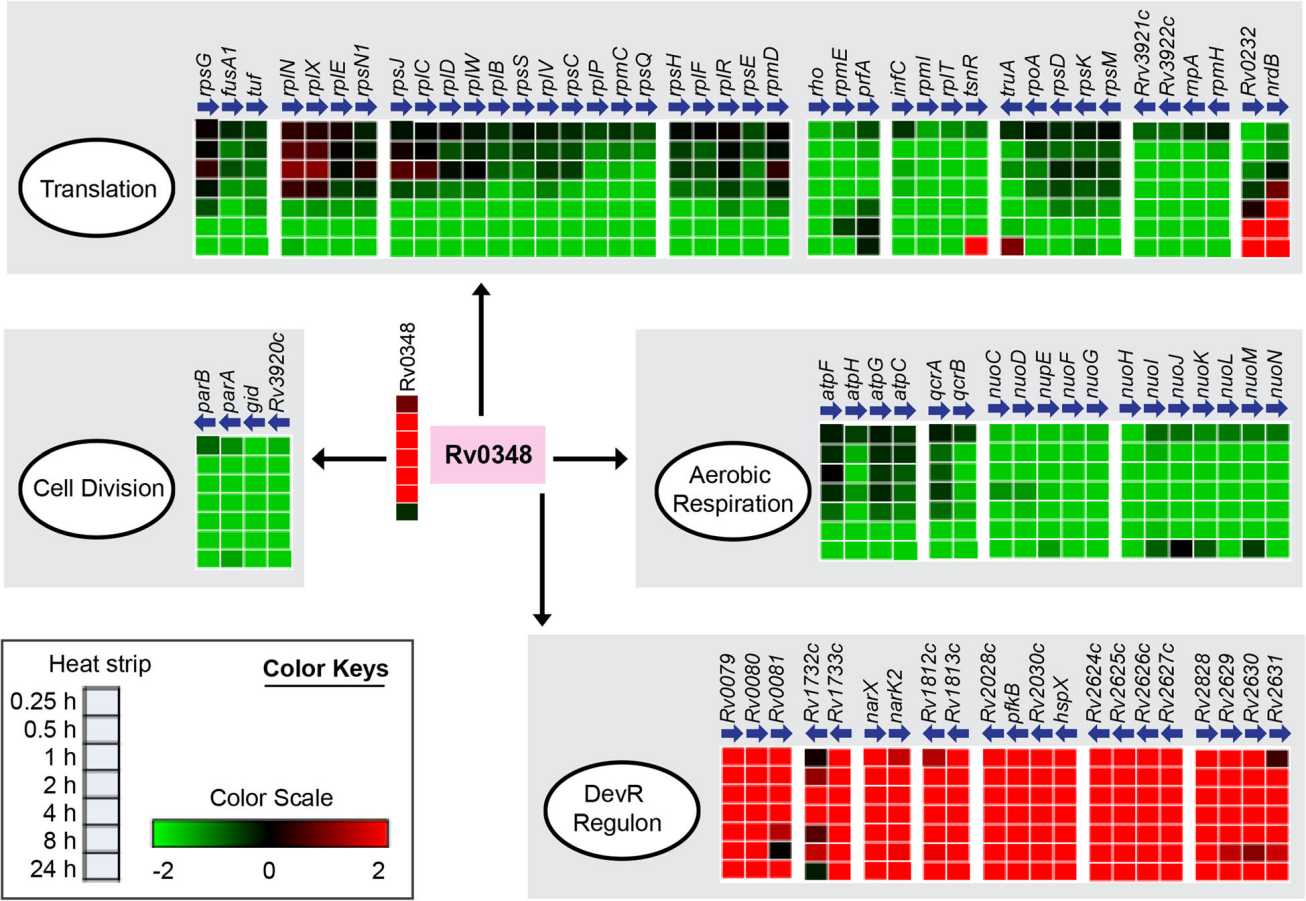
Temporal expression patterns of Rv0348 target genes. Major DRG targets of Rv0348 arranged in 4 functions with their temporal expressions is shown. Genes belonging to the same operon are arranged together and the horizontal arrows indicate the directions in which the genes are transcribed in the genome

Network analysis identified that the relative node size of Rv0348 increased over time up to 8 h with respect to other enriched regulators (Fig. 3), indicating its progressive involvement during Mtb adaptation. An examination of 176 DRG targets of Rv0348 revealed that operons involved in growth, respiration and cell division are down-regulated, while DevR dormancy regulon genes are up-regulated in vit C dormancy model (Fig. 4). Notably, Rv0348 was significantly enriched at 24 h also (*p*value = 0.041), but did not satisfy the FDR correction. Interestingly, no unique DRG targets were present for Rv0348 regulator at any of the time points, rather all of them were also targets of other regulators (Additional file 1: Table S4). Rv0348 consistently shared the maximum number of DRG targets with DevR and MprA in the temporal response (Additional file 1: Table S4), which indicated their coordinated role in regulating adaptive mechanisms in Mtb. Rv0348 is implicated in mycobacterial survival during chronic infection [52], although mechanistic details are yet to be elucidated. On the other hand, the role of DevR and MprA in mycobacterial dormancy and persistence mechanisms of Mtb is well established [53].

### DevR binding is enhanced by Rv0348 at a DevR-dependent promoter through protein-protein interactions

**Fig. 5:**
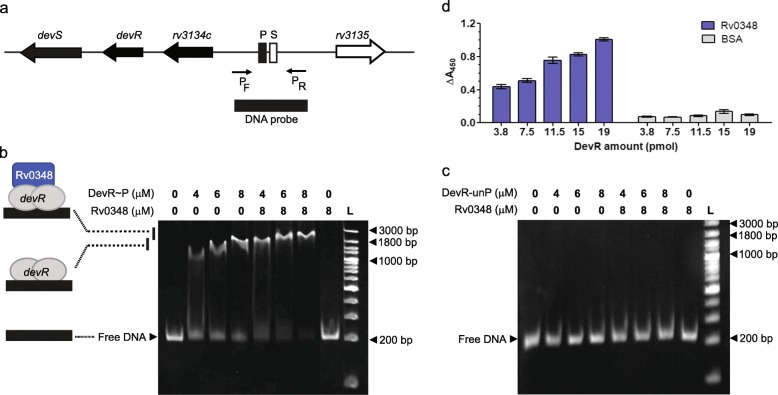
DevR and Rv0348 co-regulate DevR-dependent genes. **a** *Rv3134c* promoter region. *Rv3134c*-*devR*-*devS* operon genes are depicted by black arrows and the promoter region consists of primary (P) and secondary (S) Dev boxes where DevR binds to the promoter. The DNA probe for EMSA depicted by the black box was generated from the promoter region by PCR amplification. **b** EMSA of the *Rv3134c* promoter fragment in the presence of Rv0348 and phosphorylated DevR (DevR~P). Formation of the complex of DevR alone with DNA and a higher molecular weight complex of Rv0348, DevR and DNA are depicted on the left. **c** EMSA of the *Rv3134c* promoter fragment in the presence of Rv0348 and unphosphorylated DevR (DevR-unP). **d**. Interaction of DevR-unP (3.8 to 19 pmol) and Rv0348 (15 pmol) proteins demonstrated by ELISA. BSA was used as a negative control in the assay

DevR mediates adaptation to various intracellular stresses such as hypoxia, nitric oxide, carbon monoxide and vit C [32, 53]. It was also found to modulate host adaptive responses associated with persistent infection in the macaque model of TB [54]. Rv0348 was previously reported to possibly regulate the expression of a large number of genes through indirect mechanism [39] and only three direct targets of Rv0348 were identified from ChIP-seq data analysis [17]. In view of the predicted co-regulatory role of Rv0348 at DevR target promoters in vit C-treated cells (Additional file 1: Table S4), we investigated Rv0348 protein binding to the *Rv3134c* promoter (DevR-dependent dormancy operon promoter, Fig. 5a) in the presence and absence of DevR protein. Rv0348 did not bind to the DNA fragment at up to 8 μM of protein, ruling out the possibility of direct target regulation at DevR-dependent promoters. On the contrary, co-incubation of Rv0348 and phosphorylated DevR (DevR~P, 4 to 8 μM) resulted in a supershift of DNA (Fig. 5b). The specificity of the Rv0348-DevR~P-DNA complex was established by the failure of unphosphorylated DevR or unphosphorylated DevR + Rv0348 proteins to bind to the *devR* operon promoter (Fig. 5c). These findings were consistent with a previous report that phosphorylation of DevR is necessary for protein binding to its own promoter [55].

In the absence of direct DNA binding of Rv0348 and the occurrence of a supershift when Rv0348 and DevR were added together in an EMSA reaction (Fig. 5b), we examined the interaction of these proteins. We observed a concentration dependent increase in binding of DevR to Rv0348 (Fig. 5d). It is hence interpreted that although Rv0348 can bind to DevR independent of its phosphorylation status (Fig. 5d), the formation of a specific DNA-DevR-Rv0348 complex is determined by the phosphorylation of DevR.

These findings established the occurrence of interaction between DevR and Rv0348 regulators and suggested that this interaction could be the basis for an additional regulatory mechanism at DevR target genes.

## Discussion

An updated Mtb transcriptional regulatory network (Mtb-TRN) having 178 regulators with 3448 nodes and 16,040 interactions was constructed using the available literature. We have previously reported that axenic and intracellular Mtb acquire a dormancy phenotype upon treatment with vit C activating a pleiotropic stress response in Mtb [10, 32]. Here we have analysed the temporal transcriptome data generated previously from vit C-treated Mtb cultures [32] to decipher the transcriptional regulators underpinning the robust bacterial response in this dormancy model. This expression data was overlaid onto the Mtb-TRN and using Fisher’s exact test, we predicted the participation of 56 regulators in modulating a broad and integrated response of Mtb to vit C multi-stress environment.

Notably, 13 of these regulators were previously shown to be associated with bacterial responses to various stresses such as hypoxia (DevR, Rv0348, Rv0081, NadR, FurB and WhiB3) [13, 14, 39, 56, 57], pH stress (PhoP) [58], oxidative and nitrosative stresses (DevR, WhiB3, OxyS and sigma factors SigE, SigH and SigB [3, 23, 47, 49, 59], nutrient stress (Rv0348, RegX3, RelA, SigB and SigE) [2, 38, 39], envelope stress (MprA, SigB and SigE) [24, 60] and metabolic stress (WhiB3) [57]. We observed an overlap between regulators which were previously implicated in adaptation to individual stresses on one hand and, to vit C on the other. These findings point towards the utility of the vit C model to decipher regulatory circuits during Mtb adaptation to dormancy. Interestingly, as many as 21 regulators were identified in the vit C dormancy model which have not been studied or characterized previously, suggesting the participation of additional regulators in the Mtb stress response.

**Fig. 6:**
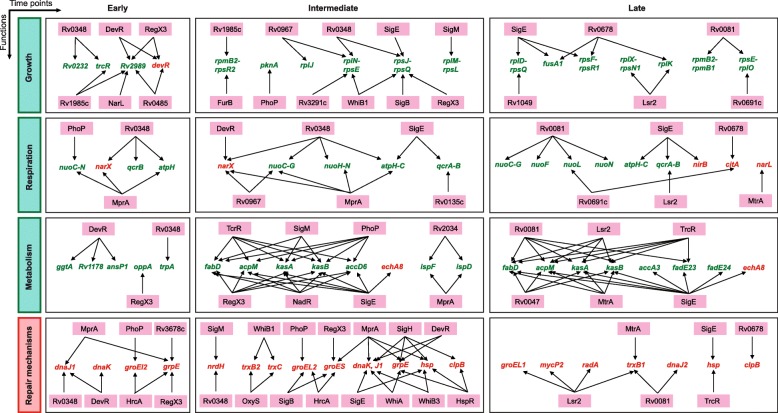
Summary of vit C-Mtb TRN involving multiple regulators and target genes. Regulators and their differentially regulated target genes arranged in 4 functions are shown. The regulators are written in pink box, up-regulated gene targets are written in red coloured font and the down-regulated gene targets are shown in green coloured font

An important finding that emerged from the analysis of Mtb-TRN was that several physiological functions (growth, respiration, repair pathways etc.) that were targeted by the enriched regulators identified in this study (Fig. 6) were also determined experimentally to be involved in the bacterial response to vit C in our previous study [32]. We found that multiple regulators were involved in the down-regulation of genes belonging to growth, respiration, and transport and metabolism of lipid, proteins and nucleotides and up-regulation of genes belonging to repair mechanisms (Fig. 6). Enrichment analysis was followed by network analysis which revealed that Rv0348, DevR and MprA were consistently identified as hub regulators having the highest number of connections in the vit C Mtb-TRN from 0.25 h to 8 h.

A completely different set of regulators was enriched at the Late time point of 24 h. Among these, Lsr2, Rv0081 and Rv0678 had the highest number of connections with the target genes in the network. The number of DRG targets increased steeply from 644 at 8 h to 1777 at 24 h, of which 1416 DRGs were unique to the 24 h time point. The expansion in the expression network at 24 h can be attributed to these Late time point regulators that orchestrate bacterial adaptation response to vit C.

Rv0348, which is a prominent hub regulator in the vit C Mtb-TRN, was previously reported as an important regulator during the chronic stage of TB in the murine infection model [52]. This regulator was shown to be conserved in the Mtb complex and modulate the transcriptional profile of ~ 163 genes [39]. Analysis of the vit C Mtb-TRN suggests that Rv0348 represses the operons related to aerobic respiration, translation and cell division. As described previously, the repression of these aerobic growth-related genes leads to growth stasis of Mtb in response to the multiple stresses imposed by vit C [32]. Out of 56 enriched regulators in the vit C Mtb-TRN, we found a maximum overlap of Rv0348 targets with DevR targets. Although several genes were reportedly under regulation by Rv0348, its direct binding to these gene targets was not observed [39]. In the present study, we established that Rv0348 physically interacts with DevR protein to enhance the binding of DevR to its target DNA. Therefore our data suggests that protein-protein interaction between Rv0348 and other regulators such as DevR can serve as an additional regulatory strategy employed by Rv0348 to orchestrate extensive gene regulation during adaptation to a multiple stress environment conferred by vit C.

Overall, our data suggests that the enriched regulators exhibited only a minor or no change in gene expression (62.5% of enriched regulators had no change in gene expression at 5 out of 7 time points, Additional file 1: Table S5), whereas there were extensive changes in the expression of their target genes. The extensive gene modulation by these regulators can be attributed to the activation of the regulator proteins, for example by phosphorylation as shown for DevR, and by interaction between regulators as exemplified by DevR-Rv0348 interaction.

## Conclusion

There is involvement of multiple regulators and target genes during Mtb adaptation to vit C-induced dormancy. Rv0348 along with DevR and MprA are predicted to function as hub regulators in the vit C Mtb-TRN, wherein the activity of Rv0348 is likely to be mediated majorly through its interactions with other transcriptional regulators.

## Methods

### Experimental design and microarray data analysis

Independent triplicate cultures of Mtb H37Rv were exposed to 10 mM vit C for 0.25, 0.5, 1, 2, 4, 8 and 24 h. RNA was isolated from these cultures and microarray was performed. Briefly, Cy3 labeled samples were hybridized to Mtb arrays (Agilent custom 8 × 15 K), scanned and the data were extracted using Feature Extraction Software (GEO accession no: GSE101048 [32]). This data was normalized using 75th percentile shift and the change in fold expression was calculated with respect to 0 h (untreated control) using GeneSpring GX (version 13.1) software from Agilent Technologies. A total of 3967 genes were analysed and genes with at least 2-fold change in expression (induced or repressed) with *p*value (Student’s t-test) and false discovery rate (FDR) corrected (Benjamini and Hochberg method) *p*value ≤ 0.05, were considered to be differentially regulated genes (DRGs).

### Construction of updated Mtb transcriptional regulatory network

Firstly, we compiled the available data from multiple sources which included inference from orthology with *Escherichia coli* (*E. coli*) and *Corynebacterium glutamicum* [15, 16], DNA-binding studies [15, 17] and operon-based studies [15, 16] into a Mtb transcriptional regulatory network (Mtb-TRN, Fig. 1a). The network was further updated by including all literature reports from June 2012 to August 2018 and then an operon-based expansion was performed [33], considering the fact that transcription factors (TFs) binding to the promoter region would regulate all the genes in the operon as previously described [14].

### Enrichment of regulators

Regulators were enriched on the basis of their target genes that were differentially expressed in response to vit C. Considering that TFs can mediate the expression of the downstream target genes by post-translational modification such as phosphorylation, while maintaining relatively constant mRNA expression levels, the role of regulators in Mtb dormancy was determined only by analysing the expression of their target genes [14, 34]. Briefly, the statistical significance of the association between the target set of every regulator and the entire DRG set in the vit C model of Mtb dormancy was evaluated by applying Fisher’s exact test (FET) for each time point (as done by [35]). The *p*values obtained were subjected to false discovery rate (FDR) analysis using Benjamini and Hochberg method and a regulator with FDR corrected *p*value of ≤0.05, was considered to be enriched (Fig. 1a).

### Functional enrichment analysis

A functional enrichment analysis was performed on the DRG targets (up-regulated and down-regulated pooled together) of all the enriched regulators using FET and the genes were classified into the functional sets defined by Cluster of Orthologous Genes (COG) database. A functional class with a *p*value of ≤0.05 was considered significantly enriched.

### Network analysis and visualization

Networks were constructed for every time point using Cytoscape software (Version 2.0) [36] which consisted of the enriched regulators and their DRG targets for that particular time point. We used Cytoscape’s NetworkAnalyzer function for analysing the network which computes different network statistics including degree, betweenness centrality and closeness centrality. The networks were visualized in Cytoscape using node degree scores.

### RNA isolation and reverse transcriptase-qPCR

Mtb was cultured in DTA medium (Dubos medium containing 0.5% BSA, 0.75% Dextrose and 0.085% NaCl plus 0.1% Tween-80) as 10 ml cultures in 50 ml tubes with shaking at 220 rpm till an OD_595_ ~ 0.1–0.2 and treated with 10 mM vit C from 0.25 h to 24 h with 0 h untreated cultures as control. RNA was isolated from the treated and untreated cultures using TRIzol based isolation method as described previously [10]. Total bacterial RNA (500 ng) was reverse transcribed to cDNA and analysed by qPCR using gene specific primers (Table 1) as described [10]. Fold change in gene expression in vit C treated versus 0 h untreated cultures is represented after normalizing the transcripts with respect to 16S rRNA.

**Table 1:** Primers used in the study

Primer	Sequence (5′➔3′)	Assay
16S rRNA F 16S rRNA R	ATG ACG GCC TTC GGG TTG TAA CGG CTG CTG GCA CGT AGT TG	RT-qPCR
Rv0348 F Rv0348 R	AGC TCG CGG ACT ACG GCT TT GGT TCT CGC CGG TAA CTC CA	RT-qPCR
devR F devR R	CCG ATC TGC GCT GTC TGA TC GTC CAG CGC CCA CAT CTT T	RT-qPCR
mprA F mprA R	GAC CTG CCG ATT CTG GTG CT GGC GAA CGG CTT TGG TAG GT	RT-qPCR
regX3 F regX3 R	TGA CGA CTA CGT GAC CAA GC CTC ATC TCC GAG TCG TCG TC	RT-qPCR
lsr2 F lsr2 R	GGT CGA ATT CGG GCT TGA CG GCC ACC CAT TGC TTC AGG TC	RT-qPCR
Rv0081 F Rv0081 R	CCT GGA GTC GTC GAA CCT GT GGG TGC GGC AAT CGA ATA GA	RT-qPCR
Rv0678 F Rv0678 R	GCT GGC TGC TGG TGT GTG AT GGA TCA GCA TCC GGG CAT T	RT-qPCR
mosRNcoI F mosRXhoI R	GCCGCCCCATGGCCATGACCATTTCGTTCTCTAGC GCCGCCCTCGAGCCGCTTGGGTCTTATCGAGATC	Cloning
Rv3134c Up F Rv3134c Up R	GAC GGC CGC TGG TTC GGC AG TTA CTG GCG GGG ACC GCT ATC T	EMSA

The cloning sites inserted in the primers are underlined

### Cloning and purification of Rv0348-His_6_ from *E.coli*

*Rv0348* was amplified from Mtb H37Rv genomic DNA and cloned in *E. coli* expression vector, pET28a at NcoI and XhoI restriction sites to obtain the C-terminally His-tagged Rv0348. The sequence of primers used for cloning are given in Table 1. This was followed by transformation of the recombinant vector into competent *E. coli* DH5α cells. After sequence verification, Rv0348-His_6_ protein was over-expressed in *E. coli* C43 (DE3) grown at 37 °C with shaking at 180 rpm till OD_595_ of 0.4–0.5 in 2x YT media containing Kanamycin (50 μg/ ml) followed by induction with 1 mM IPTG. Protein was purified by lysing the bacterial inclusion bodies and refolding the protein on the Ni-NTA column as described previously [37].

### Electrophoretic mobility shift assay

Mtb H37Rv genomic DNA was used as template to generate DNA probe for the *Rv3134c* promoter region (*devR* operon promoter). The primers used for amplification of probes from genomic DNA are given in Table 1. In a standard Electrophoretic mobility shift assay (EMSA) reaction, Rv0348 was incubated with 250 ng DNA in buffer containing 25 mM Tris-HCl (pH 8.0), 0.5 mM EDTA, 0.5 mM DTT, 20 mM KCl and 5% glycerol for 30 min on ice in a final reaction volume of 20 μl. For super-shift EMSAs, varying concentrations of His_6_-DevR protein were phosphorylated using 50 mM acetyl phosphate in buffer containing 25 mM Tris -HCl (pH 8.0), 0.5 mM EDTA, 20 mM KCl, 6 mM MgCl and 2.5% glycerol for 25 mins at 25 °C. Phosphorylated DevR (DevR~P) was then incubated with *devR* operon promoter fragment (*Rv3134c*; Table 1) in the presence of Rv0348-His_6_ protein for 30 mins on ice. The reaction was electrophoresed on a 5% non-denaturing gel at 120 V in 0.5x Tris-Borate-EDTA buffer at 4 °C after pre-running the gel for 30 min and then stained with ethidium bromide for visualization.

### Enzyme linked Immunosorbent assay for assessing protein-protein interaction

Rv0348-DevR protein interaction was assessed in an Enzyme linked immunosorbent assay (ELISA) format for assessing protein-protein interaction. Briefly, 96-well microtiter plate was coated with Rv0348-His_6_ (15 pmol) protein in coating buffer (sodium carbonate-bicarbonate buffer, 0.05 M, pH 9.6) and incubated overnight at 4 °C in a humidified chamber. Control wells were coated with bovine serum albumin (BSA) or left uncoated. Blocking was carried out for 90 mins at 37 °C with 4% BSA made in 1x phosphate buffer saline (PBS) and 0.25% Tween-20 following which the plate was washed with 1x PBS. Varying concentrations of the DevR (unphosphorylated; 3.8, 7.5, 11.5, 15, 19 pmol) were added to the wells and incubated at 25 °C for 90 mins. After washing with 1x PBS, polyclonal anti-DevR antibody (generated in rabbit) was then added to each well at a dilution of 1: 5000 for 1 h at 25 °C. The plate was washed rigorously 5 times with 1x PBS and 0.1% Tween-20 and twice with 1x PBS, followed by addition of goat anti-rabbit IgG-HRP conjugate secondary antibody (1:10000) for 1 h at 25 °C. The plate was again thoroughly washed and developed with TMB substrate with absorbance measurement at 450 nm.

## Supplementary information

**Additional file 1: Table S1.** *Mycobacterium tuberculosis* transcriptional regulatory network (Mtb-TRN). **Table S2.** Details of the enriched COG functions with the genes and their regulators. **Table S3.** Temporal network analysis scores obtained using Cytoscape’s NetworkAnalyzer function. **Table S4.** List of Shared DRG targets of Rv0348 with other enriched regulators at each time point. **Table S5.** Temporal gene expression data of 56 enriched regulators.

**Additional file 2: Figure S1.** Hierarchical clustering of all differentially regulated genes in the vitamin C Mtb dormancy model. **Figure S2.** COG functional classification analysis of the differentially regulated gene targets of enriched regulators at 24 h. **Figure S3.** Temporal expression of selected top scoring nodes (regulatory genes) of network analysis from microarray and RT-qPCR analysis.

## Data Availability

All data and materials described in this publication are included in the manuscript and additional supporting files.

## Abbreviations

COG: Cluster of Orthologous Genes
DRGs: Differentially regulated genes
DTA: Dubos containing BSA, Dextrose, NaCl and Tween-80
*E. coli*: *Escherichia coli*
ELISA: Enzyme linked immunosorbent assay
EMSA: Electrophoretic mobility shift assay
FDR: False discovery rate
FET: Fisher’s exact test
Mtb: *Mycobacterium tuberculosis*
Mtb-TRN: Mtb-Transcriptional Regulatory Network
RT-qPCR: Reverse transcriptase quantitative polymerase chain reaction
TB: Tuberculosis
TFs: Transcription factors
VBNC: Viable but non-culturable
vit C: Vitamin C
vit C-Mtb TRN: Vitamin C Mtb TRN
