# Supplementary material for: Multiple transcription factors co-regulate the Mycobacterium tuberculosis adaptation response to vitamin C
Source: BMC Genomics. 2019 Nov 21;20:887. doi: 10.1186/s12864-019-6190-3 (PMC6868718; doi:10.1186/s12864-019-6190-3)
Supplement: Supplementary file 2 — Additional file 2: Figure S1. Hierarchical clustering of all differentially regulated genes in the vitamin C Mtb dormancy model. Figure S2. COG functional classification analysis of the differentially regulated gene targets of enriched regulators at 24 h. Figure S3. Temporal expression of selected top scoring nodes (regulatory genes) of network analysis from microarray and RT-qPCR analysis. [file 12864_2019_6190_MOESM2_ESM.docx]

**Additional File 2**

**Multiple transcription factors co-regulate the *Mycobacterium tuberculosis* adaptation response to Vitamin C**

Malobi Nandi^1,2^, Kriti Sikri^1^, Neha Chaudhary^1,$^, Shekhar Chintamani Mande^3^, Ravi Datta Sharma^2^, Jaya Sivaswami Tyagi^1,4,*^

^1^Department of Biotechnology, All India Institute of Medical Sciences, New Delhi 110029, India; ^2^Amity Institute of Biotechnology, Amity University, Manesar, Haryana 122413, India; ^3^National Centre for Cell Science, Pune, Maharashtra 411007, India; ^4^Translational Health Science and Technology Institute, Faridabad, Haryana 121001, India.

**
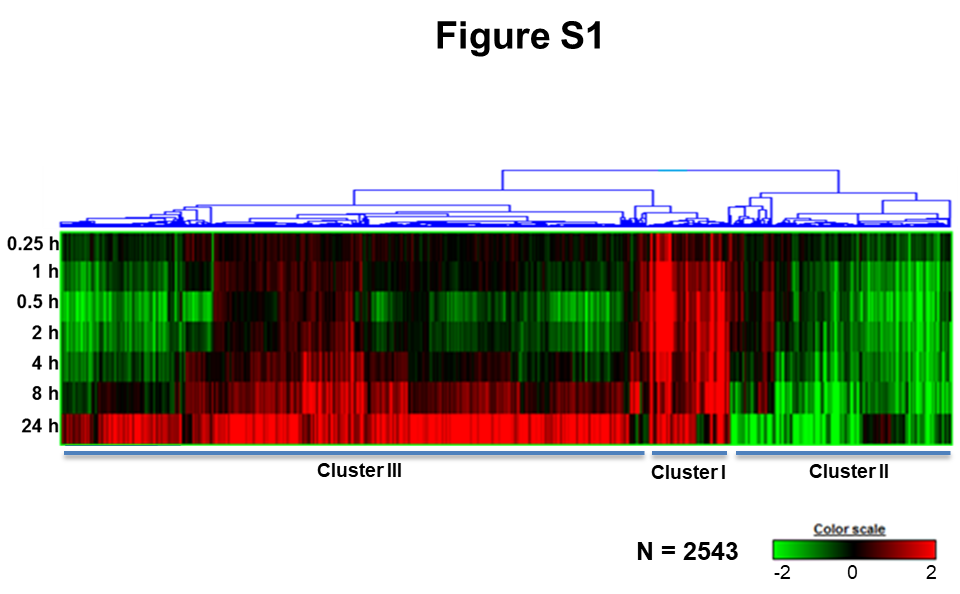
Figure S1**. Hierarchical clustering of all differentially regulated genes in the vitamin C Mtb dormancy model.


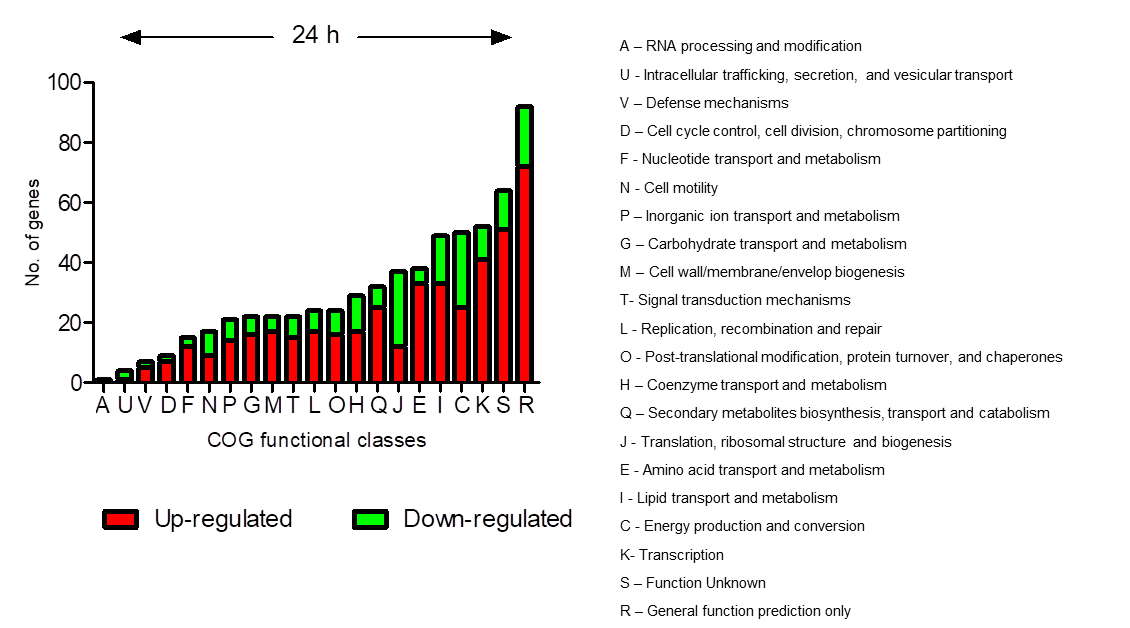


**Figure S2.** COG functional classification analysis of the differentially regulated gene targets of enriched regulators at 24 h.


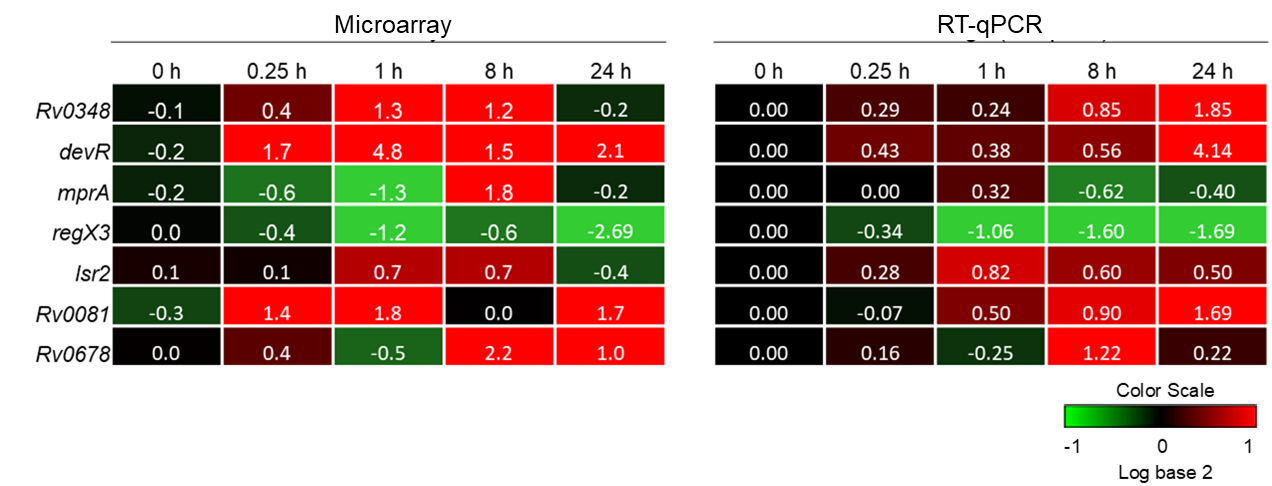


**Figure S3. Temporal expression of selected top scoring nodes (regulatory genes) of network analysis from microarray and RT-qPCR analysis.** Fold change is calculated for vit C-treated cultures from each time point versus untreated culture at 0 h and average values from 3-4 replicates are shown.
